# Supplementary material for: De Novo Hydration of Cryo-EM Reconstructions through Molecular Dynamics Simulations of the Excess Chemical Potential
Source: bioRxiv. 2025 May 23:2025.05.23.655847. Preprint. [Version 1] doi: 10.1101/2025.05.23.655847 (PMC12139794; doi:10.1101/2025.05.23.655847)
Supplement: Supplement 1 [file media-1.pdf]

Supplementary Material

De Novo Hydration of Cryo-EM Reconstructions through Molecular Dynamics Simulations of the Excess Chemical Potential

Table S1 Summary information of 9 cryo-EM structures of apoferritin (Group A) with resolution higher than 1.5 Å and 17 cryo-EM structures of apoferritin (Group B) with resolution between 1.5 and 2.0 Å.

PDB ID	Year of Deposition	Resolution (Å)	Refinement Software	Number of Cryo-EM Water per Chain
**Group A (9)**				
8RQB	2024	1.09	Unknown	110
7A6A	2020	1.15	Refmac	194
8J5A	2023	1.19	Refmac	145
7A4M	2020	1.22	Refmac	110
6Z6U	2020	1.25	Refmac	139
7RRP	2021	1.27	Phenix	102
7A6B	2020	1.33	Refmac	162
7K3V	2020	1.34	Phenix	111
7K3W	2020	1.36	Phenix	111
**Group B (17)**				
6Z9E	2020	1.55	Refmac	146
6Z9F	2020	1.56	Refmac	172
7R5O	2022	1.58	Refmac	139
7KOD	2020	1.65	Phenix	73
8EMQ	2022	1.66	Phenix	124
8EN7	2022	1.68	Phenix	106
6V21	2019	1.75	Phenix	75
8CPU	2023	1.76	Phenix	73
8CPV	2023	1.76	Phenix	71
8CPX	2023	1.76	Phenix	70
7ZG7	2022	1.77	Phenix	111
8CPT	2023	1.79	Phenix	79
8CPW	2023	1.79	Phenix	78
8CPM	2023	1.81	Phenix	81
8CPS	2023	1.82	Phenix	84
6S61	2019	1.84	Phenix	160
7VD8	2021	1.96	Phenix	103

Table S2 The observation of high- and low-consensus waters in cryo-EM maps across various resolutions (1.34 Å, 1.49 Å, ... 3.84 Å) and at three different false discovery rate (FDR) thresholds: 0.1%, 1%, and 5%.

Water Confidence	Resolution (Å)									
	1.34	1.49	1.53	1.6	1.67	1.82	2.21	2.68	2.92	3.84
**lower**	No	No	No at 0.1%, partial overlap at 1 and 5%	No at 0.1 and 1%, partial at 5%	No at 0.1%, partial overlap at 1 and 5%	No	No	No	No	No
**lower**	No at 0.1 and 1%, partial overlap at 5%	No at 0.1%, partial overlap at 1 and 5%	No	No	No	No	Noisy and partial at 0.1%, noisy at 1 and 5%	Noisy and partial	No	No
**lower**	Yes	Partial	Partial at 0.1%, complet e at 1 and 5%	No at 0.1%, partial at 1%, complet e overlap at 5%	Partial	No at 0.1%, partial overlap at 1 and 5%	No	Partial at 0.1%, noisy at 1 and 5%	Noisy and partial at 0.1%, noisy at 1 and 5%	No
**lower**	No at 0.1%, partial at 1 and 5%	No at 0.1 and 1%, partial overlap at 5%	No	No at 0.1%, partial at 1 and 5%	No	No at 0.1%, partial overlap at 1 and 5%	No at 0.1 and 1%, partial overlap at 5%	No	No	No
**lower**	No	No	No	No	No	No	No	No	No	No
**lower**	Partial	Partial	No at 0.1%, partial overlap at 1 and 5%	No at 0.1%, partial at 1 and 5%	No at 0.1%, partial overlap at 1 and 5%	No at 0.1 and 1%, partial overlap at 5%	No at 0.1%, partial and noisy at 1 and 5%	No	No	No
**lower**	No at 0.1 and 1%, partial overlap at 5%	No at 0.1%, partial overlap at 1 and 5%	No	No at 0.1%, partial at 1 and 5%	No at 0.1 and 1%, partial overlap at 5%	Partial	No at 0.1%, partial overlap at 1 and 5%	No	No	No
**lower**	No at 0.1%, partial at 1 and 5%	No	No	No	No	No at 0.1%, partial overlap at 1 and 5%	Noisy and partial at 0.1%, noisy at 1 and 5%	Noisy and partial at 0.1 and 1%, noisy at 5%	No	No
**lower**	Partial	Partial	No at 0.1%, partial overlap at 1 and 5%	No at 0.1 and 1%, noisy at 5%	No	No at 0.1%, partial overlap at 1 and 5%	No	No	No	No
**lower**	Partial	Partial	No at 0.1 and 1%, partial at 5%	No at 0.1 and 1, noisy at 5%	No	No at 0.1%, partial overlap at 1 and 5%	No	No	No	No
**lower**	Yes	Partial	Yes	Yes	Yes	Yes	Yes	Noisy	No at 0.1% and 1% and partial	No

									overlap at 5%	
**lower**	Yes	Yes	Yes	Yes	Yes	Yes	Noisy	Noisy	No	No
**lower**	Yes	Yes	Yes	No at 0.1, noisy at 1 and 5%	Yes	Yes	Yes	No at 0.1, noisy and partial overlap at 1% and 5%	No	No
**lower**	Noisy	Noisy	Yes	Yes	Yes	Yes	Noisy	Noisy and partial	No	No
**lower**	Yes	Yes	Noisy	Yes	Yes	Partial at 0.1%, complete at 1 and 5%	Yes	Yes	Yes	No
**lower**	Yes	Partial at 0.1 and 1%, complete overlap at 5%	No at 0.1%, partial overlap at 1 and 5%	Yes	No at 0.1%, partial overlap at 1 and 5%	Partial	Yes	Partial at 0.1 and 1%, noisy at 5%	No	No
**lower**	Partial at 0.1%, complete overlap at 1 and 5%	Yes	Partial at 0.1 and 1, complete overlap at 5%	Partial at 0.1 and 1%, noisy at 5%	Partial at 0.1, complete overlap at 1 and 5%	Yes	Yes	No at 0.1%, partial at 1 and 5%	No	No
**lower**	Partial at 0.1%, complete overlap at 1 and 5%	Partial	Partial	No	No	No	No at 0.1%, partial and noisy at 1 and 5%	Noisy	No	No
**lower**	Yes	Yes and elongated density	No at 0.1%, overlap at 1 and 5% with elongated density	Yes	No at 0.1%, partial overlap at 1 and 5%	Partial	Noisy	Partial at 0.1 and 1%, partial and noisy at 5%	No	No
**lower**	Partial at 0.1 and 1%, complete overlap at 5%	Yes	Partial at 0.1 and 1, complete overlap at 5%	No	No	No at 0.1%, partial overlap at 1 and 5%	Noisy	Noisy	No	No
**lower**	Yes	Yes	Partial at 0.1, complete overlap at 1 and 5%	Yes	Partial at 0.1, complete overlap at 1 and 5%	Yes	Noisy	No	No	No
**lower**	Yes	Yes	Yes	Partial	Partial overlap at 0.1%, complete overlap 1 and 5%	Yes	Yes	No	No	No

**lower**	Yes	Partial at 0.1%, complet e overlap at 1 and 5%	Yes	Partial at 0.1%, complet e overlap at 1 and 5%	Partial	No at 0.1 and 1%, partial overlap at 5%	Noisy	Noisy	No	No
**lower**	Partial at 0.1%, noisy and partial at 1%, noisy at 5%	Yes	Yes	No	No	No at 0.1%, partial overlap at 1 and 5%	Noisy	No at 0.1%, noisy and partial at 1 and 5%	No	No
**high**	Yes	Yes	Yes	Yes	Yes	Yes	Yes	Noisy	No	No
**high**	Yes	Yes	Yes	Yes	Yes	Partial overlap at 0.1 and 1%, complet e overlap at 5%	Noisy	Yes	No	No
**high**	Yes	Yes	Yes	Yes	Yes	Yes	Noisy	Yes	No	No
**high**	Yes	Yes	Yes	Yes	Yes	Yes	Yes	Noisy	Partial at 0.1%, noisy at 1 and 5%	No
**high**	Yes	Yes	Yes	Yes	Yes	Yes	Noisy	Partial at 0.1, noisy at 1 and 5%	No at 0.1%, noisy at 1 and 5%	No
**high**	Yes	Yes	Yes	Yes	Partial overlap at 0.1 and 1%, complet e overlap at 5%	Partial overlap at 0.1 and 1%, complet e overlap at 5%	Noisy	Noisy	No	No
**high**	Yes	Yes	Yes	Yes	Yes	Yes	Yes	Noisy	No at 0.1%, partial overlap at 1% and 5%	No
**high**	Yes	Yes	Yes	Yes	Partial at 0.1, complet e overlap at 1 and 5%	Yes	Noisy	Partial at 0.1, complet e overlap at 1, noisy at 5%	No	No
**high**	Yes	Yes	Yes	Yes	Yes	Yes	Yes	Partial at 0.1, complet e overlap at 1 and 5%	No	No
**high**	Yes	Yes	Yes	Yes	Yes	Yes	Yes	Noisy	No at 0.1%, partial overlap	No

									at 1% and 5%	
**high**	Yes	Yes	Yes	Yes	Yes	Yes	Yes	Noisy	No	No
**high**	Yes	Yes	Yes	Yes	Noisy	Noisy	No	No at 0.1, noisy and partial overlap at 1% and 5%	No at 0.1%, noisy at 1 and 5%	No
**high**	Yes	Yes	Yes	Yes	Yes	Yes	Yes	Noisy	No	No
**high**	Yes	Yes	Yes	Yes	Yes	Yes	Noisy	Noisy	Noisy	No
**high**	Noisy	Yes	Yes	Yes	Yes	Yes	Noisy	Noisy and partial at 0.1 and 1%, noisy at 5%	No	No
**high**	Yes	Yes	Yes	Yes	Yes	Yes	Yes	Partial at 0.1 and 1%, noisy at 5%	No	No
**high**	Yes	Yes	Yes	Yes	Yes	Yes	Noisy	Noisy	No	No
**high**	Yes	Yes	Yes	Yes	Yes	Yes	Noisy	Noisy and partial at 0.1 and 1%, noisy at 5%	No	No
**high**	Yes	Yes	Yes	Yes	Yes	Yes	Noisy	Partial at 0.1%, complet e overlap at 1 and 5%	No at 0.1%, partial overlap at 1% and 5%	No
**high**	Yes	Yes	Yes	Yes	Yes	Yes	Noisy	Noisy	No	No
**high**	Yes	Yes	Yes	Yes	Yes	Yes	Noisy	Noisy	No at 0.1 and 1%, noisy at 5%	No
**high**	Yes	Yes	Yes	Yes	Yes	Yes	Yes	Yes	No	No
**high**	Yes	Yes	Yes	Yes	Yes	Yes	Yes	No at 0.1, noisy and partial overlap at 1% and 5%	No at 0.1, noisy and partial overlap at 1% and 5%	No
**high**	Yes	Yes	Yes	Yes	Yes	Yes	Noisy	Yes	No at 0.1%, partial at 1 and 5%	No
**high**	Yes	Yes	Yes	Yes	Yes	Yes	Noisy	Noisy	Noisy	No
**high**	Yes	Yes	Yes	Yes	Yes	Yes	Noisy	Yes	Partial at 0.1, complet e	No

									overlap at 1 and 5%	
**high**	Yes	Yes	Yes	Yes	Yes	Yes	Yes	Yes	No at 0.1%, partial overlap at 1% and noisy at 5%	No
**high**	Yes	Yes	Yes	Yes	Yes	Yes	Noisy	Noisy	No at 0.1 and 1%, noisy at 5%	No
**high**	Yes	Yes	Yes	Yes	Yes	Yes	Noisy	Yes	Noisy	No
**high**	Yes	Yes	Yes	Yes	Yes	Yes	Noisy	Noisy	No	No
**high**	Yes	Yes	Yes	Yes	Yes	Yes	Noisy	Noisy	No at 0.1%, partial at 1 and 5%	No
**high**	Yes	Yes	Yes	Yes	Yes	Yes	Noisy	Noisy	No	No
**high**	Yes	Yes	Yes	Yes	Yes	Yes	Noisy	Noisy	No	No
**high**	Yes	Yes	Yes	Yes	Yes	Yes	Yes	Yes	No	No
**high**	Yes	Yes	Yes	Yes	Yes	Yes	Noisy	Noisy	No at 0.1% and 1%, noisy and partial overlap at 5%	No
**high**	Yes	Yes	Yes	Yes	Yes	Yes	Noisy	Yes	No	No
**high**	Yes	Yes	Yes	Yes	Yes	Yes	Yes	Partial	No	No
**high**	Yes	Yes	Yes	Yes	Yes	Yes	Yes	Noisy	No	No
**high**	Yes	Yes	Yes	Yes	Yes	Yes	Yes	No at 0.1, noisy and partial overlap at 1% and 5%	No	No
**high**	Yes	Yes	Yes	Yes	Yes	Yes	Noisy	Partial at 0.1%, noisy at 1 and 5%	No	No
**high**	Yes	Yes	Yes	Yes	Yes	Yes	Noisy	Noisy	No	No
**high**	Yes	Yes	Yes	Yes	Yes	Partial at 0.1 and 1%, complet e at 5%	Yes	Noisy and partial at 0.1%, noisy at 1 and 5%	No	No
**high**	Yes	Yes	Yes	Yes	Yes	Yes	Yes	Noisy	Yes	No
**high**	Yes	Yes	Yes	Yes	Yes	Noisy	Noisy	Noisy	No	No
**high**	Yes	Yes	Yes	Yes	Yes	Yes	Noisy	Noisy	No	No

**high**	Yes	Yes	Yes	Yes	Yes	Yes	Noisy	Yes	No	No
**high**	Yes	Yes	Yes	Yes	Yes	Partial at 0.1 and 1%, complete at 5%	Noisy	Yes	No	No
**high**	Yes	Yes	Yes	Yes	Yes	Yes	Noisy	Partial at 0.1%, complete overlap at 1 and 5%	No	No
**high**	Yes	Yes	Yes	Yes	Yes	Yes	Noisy	Noisy	Partial and noisy	No
**high**	Yes	Yes	Yes	Yes	Yes	Yes	Noisy	Partial at 0.1 and 1%, complete overlap at 5%	No	No
**high**	Yes	Yes	Yes	Yes	Yes	Yes	Yes	Noisy	No at 0.1%, partial at 1% and complete at 5%	No
**high**	Yes	Yes	Yes	Yes	Yes	Yes	Yes	Yes	No	No
**high**	Yes	Yes	Yes	Yes	Yes	Yes	Noisy	Yes	Noisy	No

Figure S1: Final Apoferritin maps are shown at different resolutions (1.34 Å, 1.49 Å, ... 3.84 Å), with Y168 displayed at the bottom of each respective map at a 1% FDR threshold.

Figure S2: The plot showing the number of water molecules per Apoferritin residue as a function of map resolution is based on a 0.1% (**A**) and 5% (**B**) false discovery rate (FDR) threshold.

Figure S3: The interaction energy is plotted against the distance for two hydrogen-bonded water molecules(shown in inset) in gas phase using TIP3P model. The curves for Coulombic energy, Lennard-Jones energy and the total interaction energy are shown in grey, blue and red respectively.

Figure S4: The histogram illustrates the distribution of distances between matched predicted waters and experimental waters with a 2.2 Å distance cutoff.

SI Figure S1

Final apoferritin maps at different resolutions along with the Y168 displayed at the bottom of the respective map

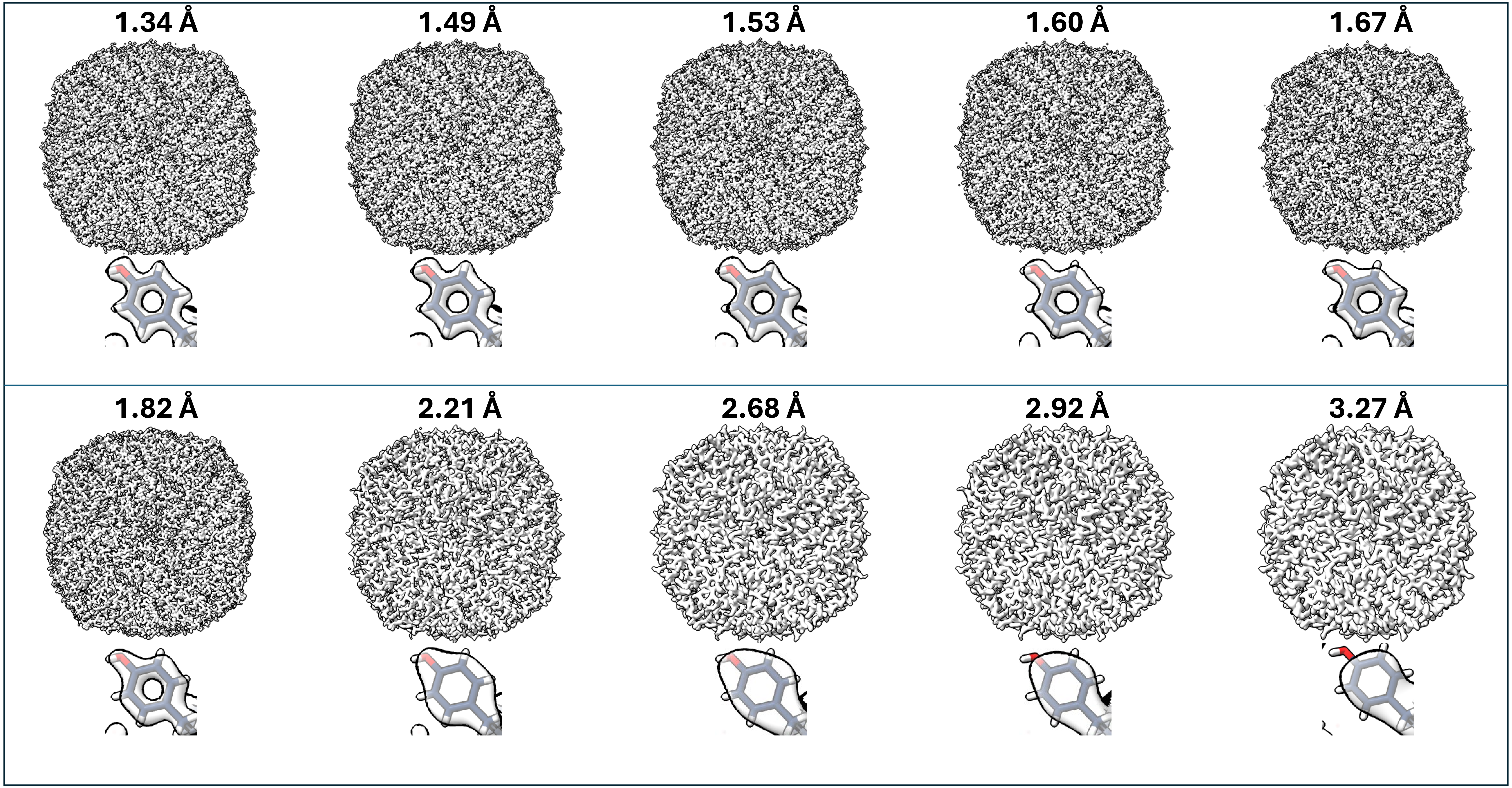

SI Figure S2

The number of water molecules per apoferritin residue as a function of map resolution at 0.1% and 5% FDR threshold

A

B

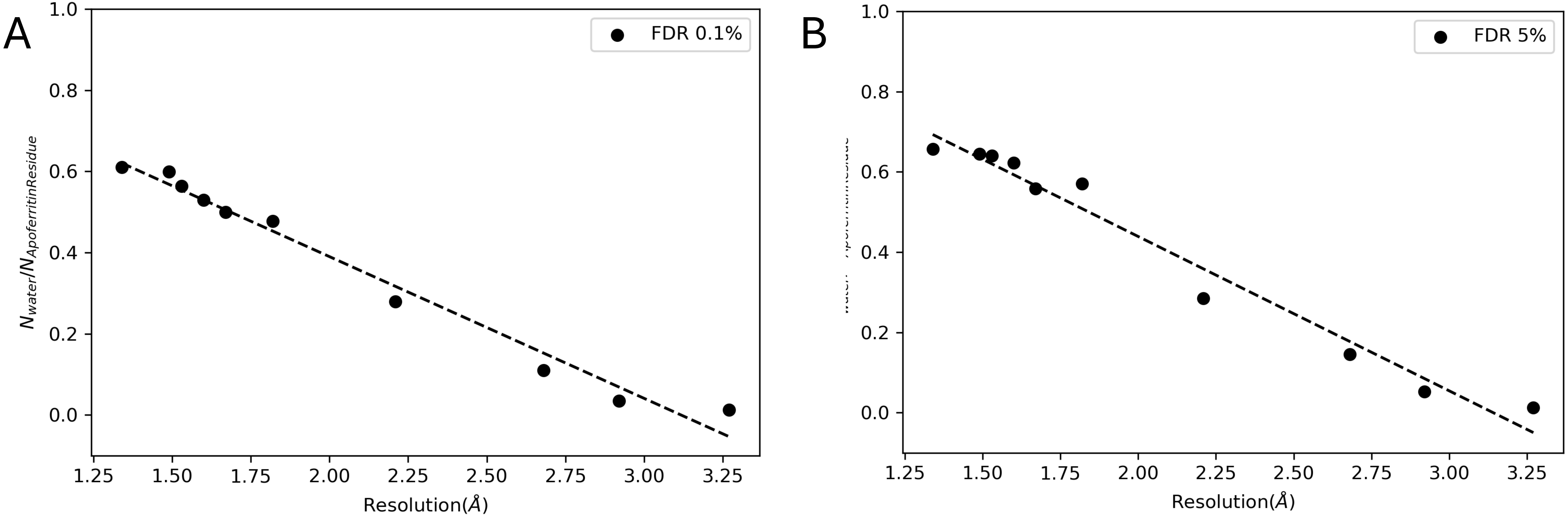

SI Figure S3

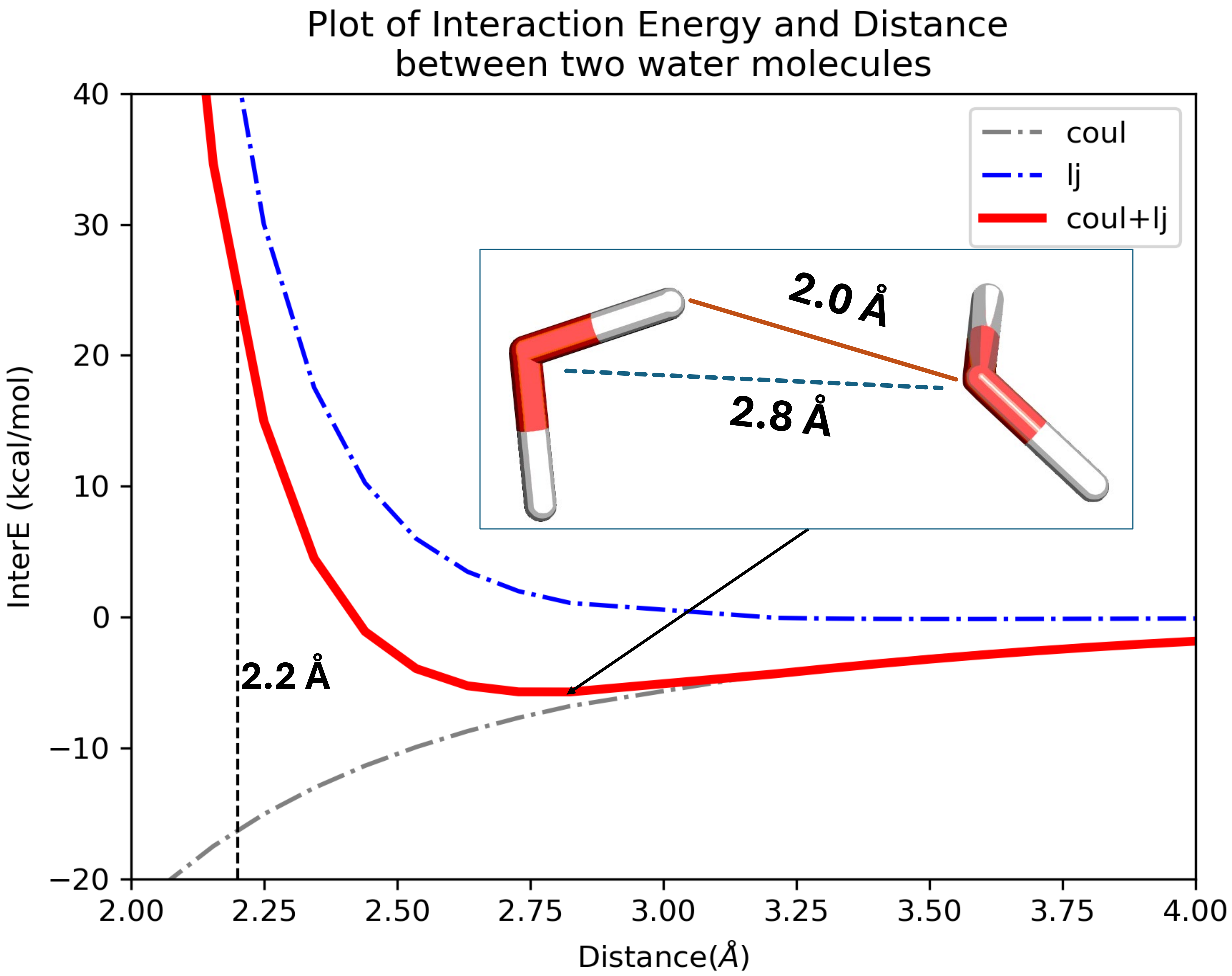

Plot of Interaction Energy and Distance
between two water molecules
InterE (kcal/mol)
Distance(Å)
40
30
20
10
0
−10
−20
2.00
2.25
2.50
2.75
3.00
3.25
3.50
3.75
4.00
coul
lj
coul+lj
2.0 Å
2.8 Å
2.2 Å

SI Figure S4

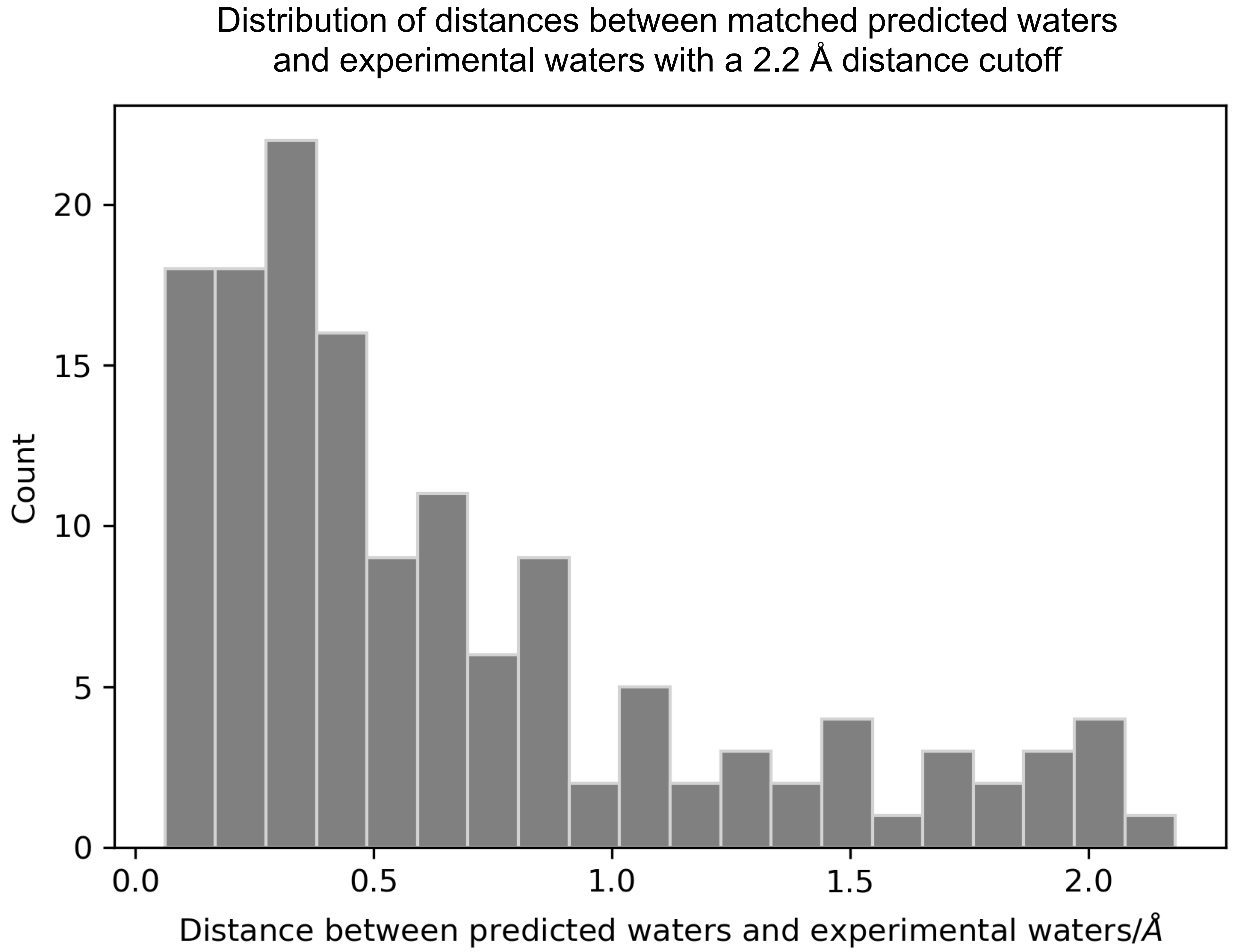

Distribution of distances between matched predicted waters
and experimental waters with a 2.2 Å distance cutoff
Count
0
5
10
15
20
0.0
0.5
1.0
1.5
2.0
Distance between predicted waters and experimental waters/Å
